# Factors affecting the preparedness of Helicopter Emergency Medical Services (HEMS) in disasters: a systematic review

**DOI:** 10.1186/s12873-023-00908-5

**Published:** 2023-11-13

**Authors:** Mahmoud Hatami, Milad Ahmadi Marzaleh, Mostafa Bijani, Mahmoudreza Peyravi

**Affiliations:** 1 Department of Health in Disasters and Emergencies, Health Human Resources Research Center, School of Health Management and Information Sciences, Shiraz University of Medical Sciences, Shiraz, Iran; 2 Department of Medical Surgical Nursing, School of Nursing, Fasa University of Medical Sciences, Fasa, Iran

**Keywords:** Preparedness, Helicopter Emergency Medical Service, HEMS, Incident, Disaster

## Abstract

**Background:**

One of the most significant advantages of Helicopter Emergency Medical Service (HEMS) in disaster relief efforts is their ability to reach inaccessible or remote areas quickly. This is especially important in the aftermath of natural disasters such as earthquakes, floods, or hurricanes, where roads may be blocked or damaged, and conventional ground transportation may not be available. There are many factors can affect the performance of Helicopter Emergency Medical Service (HEMS) in disasters. This study aims to investigate the factors affecting the Helicopter Emergency Medical Service (HEMS) in disasters.

**Methods:**

The systematic search in Cochran Library, PubMed, Scopus, Science Direct, Web of Science, ProQuest, and Google Scholar databases between the first of January in 1975 and the thirty-first of May in 2023. The articles were selected based on the keywords of the authors. At last, the criteria were extracted from the selected ones.

**Results:**

The primary search included 839 articles. After studying their title, abstract, and full context, only nine articles, including two qualitative and seven quantitative ones, were chosen for analysis. After analysis and extracting data from the final studies, the preparation factors were categorized into 6 general classes of human resources: training and practicing, management, instructions and standards, equipment, and structure. Among these, the role of training is highlighted by holding practice and maneuvers to improve and prepare the personnel and manage disasters and incidents.

**Conclusion:**

The results obtained from this systematic review provide a total view of the factors affecting the preparation of the air ambulance during disasters and incidents. It is recommended that senior managers and policy makers use the findings of the present study to identify the factors which affect preparedness of HEMS in disasters and take the necessary measures to eliminate to obstacles.

## Introduction

As a part of the Emergency Medical Service (EMS), the Helicopter Emergency Medical Service (HEMS) is one of the fastest and most efficient ways to transfer injured and sick people during emergencies, natural disasters, and road traffic incidents [1, 2]. HEMS can play a critical role in providing access to injured and sick people, transferring them from an incident scene, and ensuring their survival [3]. Today, HEMS have been developed as a part of an advanced emergency service, so many countries use these services to transfer injured and sick people during ordinary and urgent situations [1], Moreover, HEMS can transport patients quickly, saving crucial time in emergency situations. In a disaster scenario, every minute counts, and HEMS can provide rapid transport to patients who need immediate medical attention. This is particularly important for patients who require specialized care, such as trauma patients or those with severe burns, who may not be able to survive the long journey by ground transportation. [2]. In particular, HEMS presents a higher level of clinical care than road ambulance systems because they require an experienced and skilled team to cover a large geographical area. HEMS are also expensive resources that are employed to maximize clinical efficiency and operational productivity in faraway places [4]. The preparedness of the health professionals and support staff is important to dominate against disasters and protect themselves and the community from them [5]. Preparedness during emergency calls includes three main stages: emergency planning (planning/prevention); emergency reaction, saving, and recovery [6]. Preparedness is the most effective approach to managing disaster risks and is essential for emergency medical service providers such as paramedics, emergency medical technicians, and other personnel [7]. The first step to designing and developing valid tools for the evaluation of EMS providers’ preparedness against disasters as well as adopting the proper strategies for their improvement is to identify the key dimensions of this preparedness versus disasters [7]. To ensure a safe, coordinated, and efficient response, HEMS teams require the proper, proportional, and enhanced programs, which should be combined in the main planning for incidents, emergency service, and regional health care. Quick delivery of HEMS personnel with advanced skills in incident management essentials and clinical leadership is of significance to providing an efficient medical response [8]. When the incident site is farther away or the usual access to paths is dangerous, helicopters are the only way to transfer personnel and equipment to the incident scene [9]. HEMS several presumable advantages compared to ground emergency medical service (GEMS) in general. First of all, HEMS facilitate rapid preclinical patients’ transport due to increased transportation velocity [10]. Additionally, HEMS can reach the operating site irrespective of traffic and road conditions [11]. Furthermore, HEMS medical crew members are supposed to be more experienced in trauma management improving preclinical treatment of traumatized patients [10–12].

Helicopters are commonly expected to reduce the arrival time at the operating site and the transportation time from the scene to hospital due to increased velocity and the capability to avoid difficult terrain or traffic hindrance [10, 11].

HEMS units require a proper, proportional, and enhanced project for important issues including medical, air, and technical aspects. HEMS should be combined with disaster management programs and other local (non)medical emergency services, including firefighting, police, and military, to allow a cooperative and coherent response and create a strong and efficient relationship in HEMS due to mobilization and coordination of teams if a large-scale incident happens [13]. Thus, this study aims to investigate the factors affecting HEMS preparedness during disasters.

## Methods

### Eligibility criteria and search strategies

Search strategy: Searching for this systematic review is performed based on the PRISMA (Preferred Reporting Item for Systematic Reviews and Meta-analyses) guidelines [14]. To extract the related studies, a systematic search was done from the 1st of January, 1975, to the 31st of May, 2023, on the English and coordinated peer-reviewed texts associated with the research question “Which factors affect HEMS preparedness during disasters?” To verify that no systematic review had been conducted in this field, a comprehensive search was carried out using the same systematic reviews. No identical article was found in the database. Searching was done in electronic databases such as Cochran Library, PubMed, Scopus, Science Direct, Web of Science, ProQuest, and Google Scholar. Also, other sites such as the Federal Emergency Management Agency, Pan American Health Organization, World Health Organization, and Google Database were searched to find reports and guides related to the subject. Surfing the internet, reading books, theses, and conference papers were all done using gray literature. Searching between the synonymous words was performed by the “OR” operator. Then, among the word groups considered a separate concept, the “AND” operator was used. Searching for the “title, abstract, and keyword” of articles was done on different databases. On databases such as PubMed, the subject title in Mesh was used to find articles. The search strategies used in this research are shown in Table 1. In this study, a comparison group was not included in PICO. Articles and reports based on the search keywords were selected by two authors, and finally, criteria were extracted from the selected articles. In the next step, a total list was prepared from all references to papers, and their titles were explored by the researchers. Papers not related to the target were removed. All steps for searching were repeated for more reliability. The resource management was performed by EndNote software version 20.

**Table 1:** The search strategy used is based on the factors affecting HEMS preparedness in disasters

Strategy		#1 AND #2 AND #3	PIO
**#1**		Helicopter Emergency Medical Services **OR** Air Ambulance **OR** HEMS **OR** Emergency Medical Services	P
**#2**		Emergency **OR** Disaster **OR** Catastrophe **OR** Accident **OR** Incident **OR** Event	I
**#3**		Preparedness **OR** Preparation **OR** Readiness	O

### Inclusion criteria

The title and abstract of articles were selected based on this; the components of HEMS preparedness during disasters should be included in the title, keywords, or abstract, and, eventually, the entire context of the article was analyzed according to the marketing tools. Among the papers, searching for this systematic review was recovered between the first of January, 1975, and the thirty-first of May, 2023. However, the articles that had not been published (grey literature), conference papers and guidelines, protocols, instructions, and reports of valid organizations were also examined. Published in the English language or has a complete English language version available. Both quantitative and qualitative articles were selected. The search keywords existed in the title, abstract, and keyword. The articles should be associated with the research question. Also, scientific articles should be published in journals evaluated by peer review.

### Exclusion criteria

The articles referred to non-associated variables in the research question of this study was excluded.

### Screening

Two authors reviewed paper titles in databases related to study question and inclusion criteria. Selected abstracts were then read. Two authors evaluated the paper after selecting studies that met inclusion criteria. The selected papers focused on inclusion and HEMS preparedness during incidents and disasters. The evaluation of these articles was performed by the PRISMA guidelines. Meanwhile, bias was also considered in the publication, and citations and articles with high citations were accurately investigated as well.

### Data extraction

The information required was extracted from the papers based on the collected and summarized form. This form includes the corresponding author, aim and objective, population, sample, duration, design, data collection tool, methodology, results, conclusion, and the factors affecting preparedness. The summarized forms were completed for each selected paper. After completion, items of all forms were synthesized and illustrated in the descriptive tables. The authors performed this step, while others later contributed their ideas on the controversial issues. These forms were adjusted in the Word software version 2016. After studying the full text of these 9 articles, they were summarized in Table 2. The information includes title, corresponding author, study population, study sample, country, duration of the study, study design, data collection tool, methodology, results, limitations, conclusions, and factors affecting HEMS preparedness. Table 2. A summary form of selected final articles. By reviewing the literature, as well as the opinions of the research team and experts, similar factors and related to a theme were classified in one category. The results of categories and subcategories extracted from the systematic review have been shown in Table 3.

**Table 2:** A summary form of selected final articles

No.	Corresponding author/title	Aim		Population and Sample	Country and Duration	Design and Tool	Methodology	Results	Conclusion	Factors affecting the preparedness
1	Ali Sahebi, Helicopter Emergency Medical Services in 2017 Kermanshah Earthquake; a Qualitative Study		Identifying the challenges of Iran’s HEMS from the managers’ view involved in HEMS in the Kermanshah earthquake, 2017.	6 senior managers of Tehran, Kermanshah, and Ilam were involved in the Kermanshah earthquake and Managers who were directly involved in managing and transferring victims of the Kermanshah earthquake in 2016.	Iran 2018	Qualitative study using the content analysis method and Interview and Semi-structured interviews	In this qualitative/ exploratory study, 6 individual interviews were conducted with 6 managers of Iran’s HEMS to investigate what the challenges of Iran’s HEMSs are from the managers’ view during disasters, especially earthquakes.	The result revealed the following challenges: - The low priority of the preparedness program; - Lack of systematic coordination between the areas involved in the incident - Lack of a comprehensive educational program; - Limitation in infrastructure; - Disturbance in the comprehensive process of communication; - Failure to strengthen internal motivation; - Lack of skilled pilots to fly - Lack of familiarity of nurses, flight engineers, and pilots with patient transfer protocols; - Failure to maintain specialist staff.	- Necessity for implementing comprehensive training programs for the effective management of HEMS during disasters such as earthquakes; - Preparing a codified and comprehensive training program for effective and efficient management.	1- Experiences and lessons learned; 2- Compilation of air emergency preparedness plans; 3- Integrated management; 4- process-oriented preparation; 5- Infrastructure development; 6- Provision of equipment 7- Establishing a central coordination between the army, the Ministry of Health, and the Red Crescent; 8- Skilled and expert workforce access program; 9-Strengthening internal motivation; 10- How to communicate with medical teams; .
2	James Fenn, Assessment of U.S. Helicopter Emergency Medical Services Planning and Preparedness for Disaster Response		Evaluating the state of medical service planning and preparedness for disaster response in the HEMS of the United States	147 people and Association of Air Medical Services	US 2021	Quantitative and Questionnaire	This study is performed by a quantitative method through a written survey to identify problems during disasters according to the responses	9 criteria (written policy, triage, and incident command training, participation in disaster maneuvers, land, and air communications plan, critical incident stress management, annual review, and policy sharing) that significantly affect the preparedness, response, and recovery of an air medical program against disasters. Among these criteria, having a written policy is considered the most important.	A written policy that addresses key issues is essential for HEMS programs.	1- Having a written policy; 2- Development of policies; 3- Education; 4- Practicing natural disasters; 5- Zero hour exercises; 6- Review policies 7- On-time publication of policies 8- Participating in disaster practices of other organizations; 9- Stress management; 10- Incident command;
3	Karyl J. Burns, Evaluation of Responses of an Air Medical Helicopter Program during a Comprehensive Emergency Response Drill		Evaluating the responses of the HEMS program during a comprehensive emergency response exercise	4 people (an evaluation planning team) including a team leader and 3 experienced members. and Hartford Hospital Air Emergency Squad	US 2007	Quantitative and Checklist	This study is a comprehensive evaluation method for practicing developing and completing the air emergency plan. Two checklists were prepared to identify critical items.	Involvement in HEMS planning and training emergency calls has many advantages. Participating in planning an exercise familiarizes emergency management staff with the appropriate HEMS roles. It identifies requirements for developing new policies and protocols.	Evaluation of air medical emergency communications during an exercise reveals specific problems.	1- Exercises; 2- Clarification of responsibilities; 3- Implementation of instructions, protocol, and policies; 4- Planning.
4	Anne Siri Johnsen, Helicopter emergency medical services in major incident management: A national Norwegian cross-sectional survey		The objective was to describe experiences related to critical incident management and preparedness and training among all HEMS and air SAR^1^ service forces to respond to incidents.	329 people were invited and 229 responded and Doctors, pilots, air ambulance rescuers	Norway 2015	Quantitative, cross-sectional and A cross-sectional survey was conducted using a questionnaire in 3 parts of accidents, equipment, and training.	The survey consisted of 3 parts with questions related to basic demographic information, experience from real incidents, training, and equipment.	The potential to manage major incidents varies depending on the type of incident, local resources. and systems. Major events can be a challenge beyond the system’s capacity. More than half of the major incidents in Norway occurred in autumn and winter when light is low. Close collaboration in emergency response are critical.	HEMS and SAR crews are not exposed to major incident management. Interdisciplinary training takes place in repeated scenarios with a focus on collaboration and is called communication.	1- Training; 2- Research; 3- Coordination; 4- Communications; 5- Cooperation.
5	Albert Lunde, Overcommitment: Management in Helicopter Emergency Medical Services in Norway		Identifying individual approaches and organizational strategies that cause overcommitment in air SAR situations that can put both rescuers and patients at risk.	30 people And Air ambulance personnel	Norway 2019	Qualitative and Interview and focus group	In this qualitative, exploratory study, 9 focus group interviews were conducted with a total of 30 crew members from the Norwegian HEMS to investigate how air emergency personnel in Norway adjust their level of commitment in different cases.	Air ambulance personnel describe a team-based approach to adjust their level of commitment to medical evacuation and rescue missions. They focus on well-known, albeit important, non-technical skills, and organizational measures to deal with overcommitment in SAR situations.	Air ambulance personnel describe a team-based approach to adjust their level of commitment to medical evacuation and rescue missions.	1- Resource management; 2- Education; 3- Experience; 4- Standardization of training and procedures; 5- Interorganizational trainings
6	Yoshinori Okuno. Development of a helicopter Operations Management System for Disaster Relief Missions		Development of air operation management system for disaster SAR missions	110 operators and operators Air and land	Japan 2016	Quantitative, drills and System D-NET	10 operators (pilots and mission specialist crew such as air rescue teams and paramedics) evaluated the air terminals, and nearly 100 operators (dispatchers and government officers) evaluated the land terminals.	D-NET enables 3 major functions to share information essential for optimal management of helicopter operations and between them and operations bases and disaster management centers.	The air operations management system has 3 major functions: information sharing between helicopters, operational bases, and disaster management centers; Human-machine interface means internal and land terminals for entering and displaying info; and optimal operation management that can help human decision-making.	1- Inter-organizational cooperation; 2- Overcapacity management; 3- Efficient fueling and maintenance systems; 4- Coordinating the deployment of helicopters throughout the country.
7	Patryk, zońca. Experience of the Polish Medical Air Rescue Service During the First Year of the COVID-19 Pandemic and Measures Taken to Protect Patients, Medical Staff, and Air Crew from SARS-CoV-2 Infection		Presenting the experience of the Polish air medical SAR service in the first year of Covid-19 and the measures taken to protect patients, medical staff, and aircrew against SARS-CoV-2.	202 people and Patients with confirmed covid-19; The study includes a retrospective analysis of the missions of Polish air medical SAR personnel.	Poland 2021	Quantitative, retrospective analysis and Document analysis using retrospective analysis of air rescue crew missions	This study included a retrospective analysis of air-medical rescue crew missions of confirmed SARS-CoV-2 infections in Poland. The analysis included flights to accident and other emergency sites as well as patient airlift missions where medical assistance was provided to patients with confirmed SARS-CoV-2 infection between March 4, 2020, and March 4, 2021.	- Polish HEMS forces; - Using single isolation units to transfer patients; - Acute respiratory failure is the most common comorbidity in covid-19 patients; - Longer duration of out-of-hospital care causes a worse clinical condition of the patient; - Approval of the use of air transportation for patients with severe COVID-19.	This study highlights the use of single-patient isolation units for transmission.	1- Development of guidelines; 2- Personal protective equipment (PPE); 3- Patient isolation equipment during transferring.
8	Sabina Fattah. Reporting Helicopter Emergency Medical Services in Major Incidents: A Delphi Study		Research on HEMS in major incidents in Europe.	17 doctors and Prehospital intensive care physicians with previous and current experience in HEMS.	Norway 2016	Quantitative using the Delphi technique and A questionnaire using email correspondence in 5 steps	They used a Delphi approach with experts who communicated through email. The Delphi technique was used to systematically collect the opinions of a group of respondents. Questionnaires were completed repeatedly and with adjustments.	This pattern can be used as a stand-alone document to report data on the use of HEMS in pre-hospital emergency medical responses to major incidents.	Development of a consensus-based pattern for reporting air emergency responses to major incidents	1- Consensus in decisions; 2- Asking the opinion of a group of doctors; 3- Education; 4- Skilled personnel; 5- Planning.
9	Peter Hilbert-Carius, Pre-hospital care & interfacility transport of 385 COVID-19 emergency patients: an air ambulance perspective		Describe the difficulties of HEMS in Europe when transporting potential patients with COVID-19.	385 patients and COVID-19 patients in 6 European countries: Austria, Denmark, Luxembourg, Germany, Norway, Switzerland	Germany 2020	Quantitative, Retrospective and Collecting data from all missions from February 1, 2020, to April 30, 2020, and using retrospective analysis	6 European HEMS-providing countries reported their experiences and data on care, transport, and safety measures in suspected or confirmed COVID-19 missions, and 385 missions related to COVID-19 were analyzed.	Establish special safety procedures and guidelines for COVID-19 preparedness: - land transportation is the preferred method in initial missions; - Preparing air ambulance in advance for missions related to COVID-19; - Availability of care and safe transport of suspected or confirmed COVID-19 patients;	All HEMS providers were prepared for COVID-19.	1- Cleaning and disinfection of helicopters; 2- PPE; 3- Having air conditioning in helicopters; 4- Safety instructions; 5- Education.

**Table 3:** Categories and subcategories extracted from the systematic review on HEMS preparedness in disasters

Categories	Subcategories
Education and training	Natural disasters exercise
	Zero-hour exercises
	Participation in holding disaster drills in other organizations
	Training to facilitate effective interaction with others
	Research
	Inter-organizational training
	Practicing adaptation to new conditions
	Teaching how to communicate
	Teaching how to coordinate
	Teaching how to cooperate
Human resource	Experiences
	Strengthening internal motivation
	Skills
	Learned lessons
	Stress management
Management	Integrated management
	Incident command system
	Reviewing policies at least once a year
	Clarifying responsibilities
	Compilation of air emergency preparedness plans
	Coordination
	Communications
	Resource management
	Consensus in decisions
	Coordination of the helicopter deployment
	Preplanning for important events
	Overcapacity
	Having a plan to access skilled and specialized workforce
	Inter-organizational cooperation
	Land and air communication planning
	Financing
	Optimal planning
	A systematic approach to patient care
Infrastructure	Emergency operation center and air-ground traffic control center
	Establishing a central coordination center for the emergency air transport management unit between the army, Ministry of Health, and Red Crescent
	Insufficient helicopter pads in urban and intercity distances
	Systems for efficient fueling and maintenance
Instructions and standards	Safety instructions
	Implementation of guidelines, protocols, and policies
	Having a written policy
	Timely publication of policies along with their updates
	Disaster response guidelines
	Development of policies
	Educational standardization and procedures
Equipment	Having air conditioning in helicopters
	Telemedicine-based equipment
	Patient isolation devices during transferring
	PPE
	How to clean and disinfect helicopters
	Supply and maintenance of specialized equipment

## Results

**Fig. 1:**
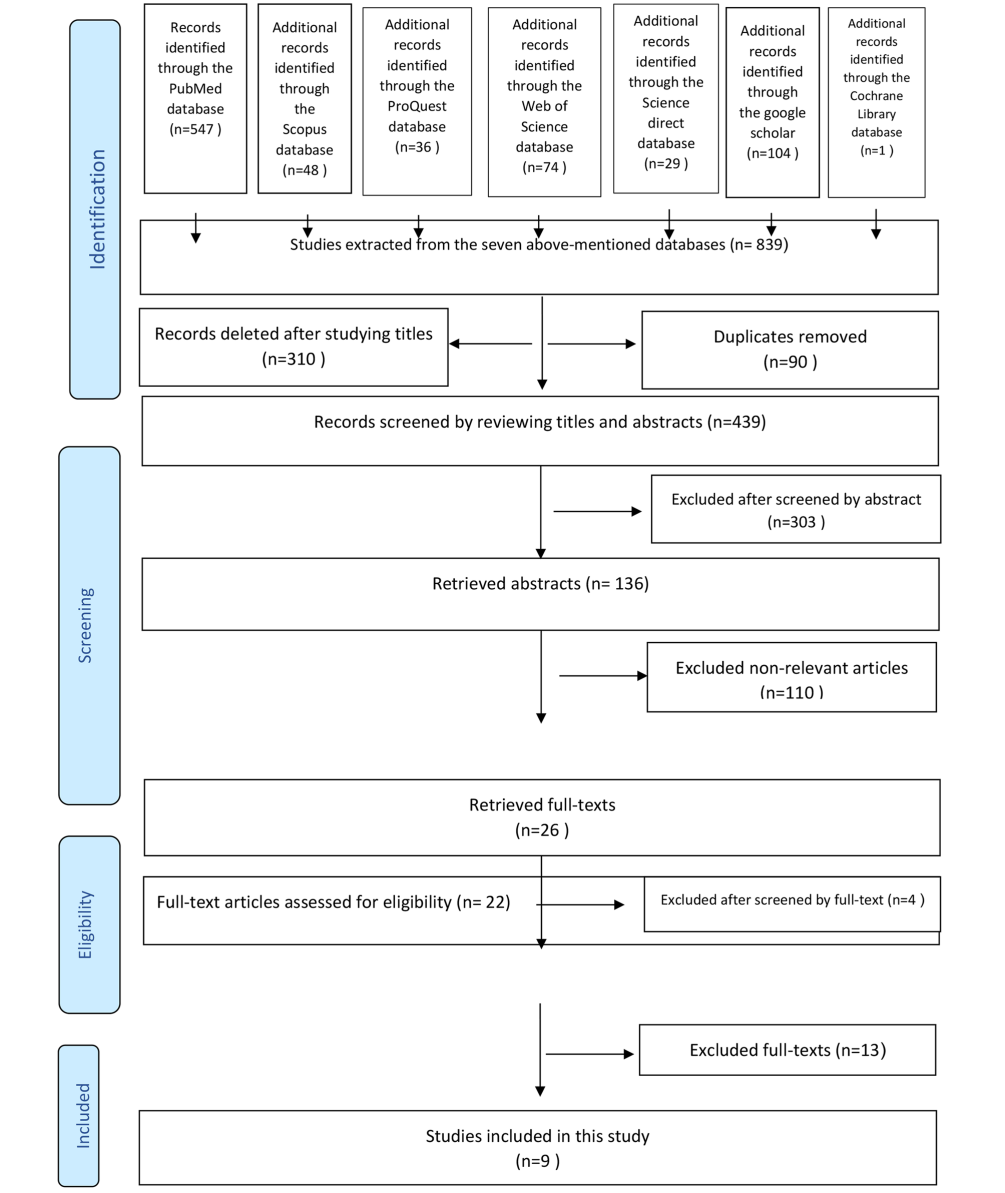
PRISMA 2020 flow diagram for new systematic reviews which included searches of databases and registers only

After searching the database, 839 papers were extracted. 90 repeated papers in different databases were removed. After investigating 749 papers, 310 were excluded because they were not in line with its aim. The abstract of 439 remaining articles from the previous step was studied and 303 were excluded due to being not associated with our aim. So, 136 full-text articles were chosen for our research, 26 were accepted, and eventually, only 9 papers were consistent with the aim of our study. Figure 1: PRISMA 2020 flow diagram for new systematic reviews which included searches of databases and registers only.

## Discussion

This study examines air ambulance preparedness criteria in different countries. Of the nine final papers, seven were quantitative and analyzed air preparedness during disasters and incidents using questionnaires and checklists. The other two papers were qualitative and utilized interviews with HEMS experts and beneficiaries. Most studies were conducted in Norway, including three. Subsequent studies were conducted in other countries, including two in the US and one each in Iran, Germany, Japan, and Poland. After analyzing and extracting data from the final studies, six categories of preparedness factors were identified: human resources, training and practice, management, instructions and standards, equipment, and structure.

### Human resources

HEMS personnel should have been adequately prepared both mentally and physically before responding to any situation. Studies suggest that emergency department personnel may experience distractions from worrying about their families during disasters, which could impact their focus and accuracy in caring for injuries. They should be able to communicate with them by telephone to be aware of their health and do their duties calmly [15]. It was also proposed to create a way to address the health and welfare of personnel’s families in all their programs during a regional incident or to timely transfer information to the responding personnel. This design relieved the anxiety of personnel and allowed them to focus on their duties [16]. HEMS is a limited and expensive resource that requires very skilled and trained personnel [15], so doing an entire scientific evaluation of its use and its potential benefits is so important and essential in incident management. The most important component of preparedness during incidents is to hold maneuvers and exercises [17].

### Training and practice

The leading role of training in the enhancement and preparedness of personnel is very important, and all the studies showed that it is one of the points to be prepared for before disasters occur. Therefore, the issue of training and practicing is one of the leading points that the staff should be trained with essential practices based on increasing their ability against disasters before their occurrence. They should at least participate in this training once a year, and managers should make arrangements to provide practice and have HEMS staff participate in it. Being aware of other people’s experiences and using their learned lessons is more effective assistance for enhancing knowledge, awareness, and preparedness for disasters. Also, awareness, competence, and knowledge of personnel related to responding to injuries or disaster conditions and safety behaviors decrease. The exercises provide opportunities for operating, which are critical for preparing HEMS. Two parameters, including practice and time, are assessed for the exercises. Participation in emergency reaction practice is important because it can allow HEMS personnel to gain confidence in doing activities that may be required for a public health emergency or mass casualty incident, including situations not covered by policies and protocols. Participating in HEMS has several advantages in terms of planning and practicing emergency reactions. Firstly, participation in the planning of an exercise can familiarize emergency management staff with proper roles in HEMS while identifying what functions cannot be suitable or which of them require the development of new policies and protocols. Emergency responders and incident commanders should know that the required service should be supported by policies and protocols. These should be clarified and communicated to all staff [18]. HEMS is a limited and costly system that needs skilled and trained staff. So, doing an entire scientific evaluation is essential to its use and its potential benefits [17].

### Management

HEMS is an integral part of the management and planning of important incidents in many countries [17]. The performance of comprehensive training programs is essential to effectively managing the HEMS process during disasters such as earthquakes through integrated management. These programs include the formulation of a written and comprehensive training program; priority of the preparedness program; coordination between the fields involved in the incident; developing infrastructure; efficiency of the triage process; attention to training rules and safety instructions and efficient training; a comprehensive communication process; attention to the supply and maintenance of human resource specialized equipment; access to the skilled and specialized workforce; awareness of experiences and learned lessons; optimal planning; writing HEMS preparedness programs to be responded during disasters; process-oriented preparedness; systematic approach for caring the patients; supply and maintenance of specialized equipment; access program to the skilled and expert workforce; enhancing internal motivation of personnel; holding educational courses of how to communicate with medical teams; preparedness to create emergency operating center and air traffic control center; skilled pilots to fly in bad conditions, familiarity of nurses; and flight engineers, and pilots with the protocols for transferring patients [19].

HEMS mostly helps with treatment, triage, patient transfer, equipment, and staff. Lack of communication and insufficient air traffic control are also challenges when responding to disasters. More training and a focus on coordination, communication, and cooperation are required. So, air operating management provides essential information to manage optimal operations between helicopters, operating bases, and disaster management centers [20]. This system has three main functions: (1) sharing information between the helicopters and disaster management centers; (2) interface between humans and machines, i.e., internal and land terminals to enter and display information; and (3) optimal operating management that can help the human decision [20].

### Equipment

Based on this, two papers about COVID-19 emphasize more PPE, gloves, repellent liquid, long-sleeved clothing or other protective clothing, eye protection and an FFP3 mask, disinfectant devices, and materials that should exist in the air ambulance department [21, 22]. PPE allows safety work in a large incident area with the dangers of a normal scene [23]. HEMS equipped with PPE is very critical when caring for patients suspected of or confirmed by COVID-19. Therefore, a suitable PPE is recommended. Cleaning and disinfecting airplanes upon arrival at their home base is so important [23]. If air ambulances and airplanes have not been disinfected as much as possible before the next mission, then healthcare providers and transferred patients are exposed to a high risk of infection. Therefore, having disinfectant equipment and facilities is very important. Also, clothing and full PPE equipment should be utilized to prevent the transformation of COVID-19 and minimize the risk of infection among medical personnel. Moreover, the crew is likely to use precautions such as isolating patients or creative solutions during inter hospital transfers and longer emergency missions. The importance of using patient isolation units has been highlighted for transferring patients between hospitals and admitting them to emergency hospitals when the COVID-19 situation is unknown [24]. The danger of transferring viruses and the total state of infection and health are due to a lack of information about the infection and limited resources on the helicopter and plane, more than the health care providers in the hospital. Hence, cleaning and disinfecting ambulances and helicopters, having proper PPE, having air conditioners in the helicopters, and having access to PPE are essential [23].

### Instructions and standards

Written policies, training for incident and triage commanders, participation in natural disaster exercises, air and land communication design, managing critical incident stress, annual addresses, and sharing surveys are all essential criteria that can significantly affect a medical program’s preparedness, reaction, and recovery in the face of disasters [16].

Protocols, guidelines, and programs for responding to disasters should be prepared and identified before these events occur to reach a comprehensive and integrated standard for treatment and dispatch because speed of action and quick medical actions are the first terms of responding to such events [15]. Access to additional information is important during these incidents. For example, the number of injured and the incident distance to the establishment of helicopters and their landing place, as well as their distance to the hospital, should be considered. Also, information resources can be obtained from the local police, firefighting, and HEMS departments.

Having the policy to overcome disasters means having a written policy based on the essential and key subjects, such as developing critical policies for preparedness during disasters as a first step; necessity for facilitating effective interaction with others; training and practicing at least once a year; zero hour exercises; reviewing policies once a year; publishing policies timely (along with updating, preparing instructions for responding to disasters, HEMS, stress management, incident command training, air and land communication program, stress management of critical incidents, and sharing policies [16].

In the structure, the most important issue is to determine the data content and format while designing the equipment for sharing information, including coordination between the establishment of helicopters around the country, efficient cooperation between organizations, reaction to the broad area disaster, systems for fueling, and efficient maintenance, frequent and unnecessary missions, crowd radio communication in the air, land, operations, and bad weather conditions [20]. And, creating helicopter pads in urban and intercity distances and reducing the high costs of air emergency service [19].

Policymakers should pay a great deal of attention to incidents and disasters because such incidents can occur whenever and wherever. Based on the costly HEMS, the government’s financial supply can resolve many problems with equipment and preparation for air emergencies. Different studies have reported relatively similar factors to explain preparedness for incidents and disasters. The results of this study can formulate a comprehensive tool to assess HEMS preparedness during disasters and incidents.

### Limitations

One limitation of this study is that it only utilized English papers.

### Implications

1) A comprehensive educational plan for effectively managing the process of air ambulances during disasters.

2) Interdisciplinary educational programs about the scenarios focus on cooperation and communication.

## Conclusion

The results obtained from this systematic review provide a total view of the factors affecting the preparation of the air ambulance during disasters and incidents. It is recommended that senior managers and policy makers use the findings of the present study to identify the factors which affect preparedness of HEMS in disasters and take the necessary measures to eliminate to obstacles.

## Data Availability

The datasets generated and/or analysed during the current study are not publicly available due to the necessity to ensure participant confidentiality policies and laws of the country but are available from the corresponding author on reasonable request.

## Footnotes

1 Search and Rescue.

## Abbreviations

HEMS: Helicopter Emergency Medical Service
EMS: Emergency Medical Services
